# Longitudinal Maternal IgG and IgA Glycosylation Profiles in Pregnancy Reveal Early Immune Alterations in Placenta-Related Complications: The Rotterdam Periconception Cohort

**DOI:** 10.1161/HYPERTENSIONAHA.126.26531

**Published:** 2026-06-26

**Authors:** Lotte W. Voskamp, Melek Rousian, Anna Daniels, Manfred Wuhrer, A.H. Jan Danser, Régine P.M. Steegers-Theunissen, Koen Verdonk

**Affiliations:** Department of Obstetrics and Gynecology, Erasmus MC University Medical Center, Rotterdam, the Netherlands (L.W.V., M.R., A.D., R.P.M.S.-T.).; Department of Internal Medicine, Erasmus University Medical Center, Rotterdam, the Netherlands (L.W.V., A.H.J.D., K.V.).; Center for Proteomics and Metabolomics, Leiden University Medical Center, the Netherlands (M.W.).

**Keywords:** glycosylation, immunoglobulins, molecular structure, preeclampsia, pregnancy, placenta

## Abstract

**BACKGROUND:**

Placenta-related complications are major contributors to maternal and neonatal morbidity. Glycosylation modulates immunoglobulin function by altering molecular structure and receptor affinity. During pregnancy, IgG (immunoglobulin G) and IgA (immunoglobulin A) glycosylation normally shift toward a more anti-inflammatory profile. We hypothesized that this adaptation is attenuated in complicated pregnancies, resulting in altered glycosylation trajectories.

**METHODS:**

Women with singleton pregnancies were enrolled in the prospective Rotterdam Periconception Cohort (2017–2018). Serum samples were collected at 9, 11, 13, 22, and 32 weeks of gestational age. IgG and IgA glycosylation was measured using liquid chromatography-mass spectrometry, and summarized into glycosylation traits (galactosylation, sialylation, fucosylation, bisection). Placenta-related complications (preeclampsia, pregnancy-induced hypertension, preterm birth, or small-for-gestational-age) were identified from medical records. Longitudinal changes and group differences in Ig glycosylation were analyzed using linear mixed-effects models with false discovery rate correction.

**RESULTS:**

Among the included 193 pregnancies, 54 were complicated by at least 1 placenta-related disorder. In complicated pregnancies, galactosylation and sialylation were consistently lower, whereas fucosylation and bisection were higher. From 9 weeks of gestational age onwards, fucosylation was significantly elevated at IgA Asn327/Asn340 and Asn205 (*Z*-score difference: 0.45–0.52; *q*=0.002–0.004 and 0.33–0.37; *q*=0.038). Bisection increased more steeply at IgG1, IgA Asn144/Asn131, and IgA Asn327/Asn340 (*q*=0.021–0.027). IgG galactosylation rose less across gestation (*q*=0.021–0.024), and IgA galactosylation and sialylation declined more sharply at Asn144/Asn131, Asn205, and Asn327/Asn340 (q=0.000–0.026).

**CONCLUSIONS:**

Pregnancies with placenta-related complications exhibit more proinflammatory glycosylation profiles. These findings suggest a potential mechanistic link between immunoglobulin glycosylation and placental dysfunction, with specific glycopeptides as possible biomarkers.

**REGISTRATION:**

URL: https://onderzoekmetmensen.nl/en/trial/57880; Unique identifier: NL-OMON57880.

**Novelty and Relevance**

**What Is New?**

This study provides a comprehensive analysis of maternal IgA and IgG glycosylation in placenta-related pregnancy complications.
Pregnancies complicated by hypertensive disorders, fetal growth restriction, or preterm birth show lower galactosylation and sialylation, indicating a proinflammatory profile similar to that seen in cardiovascular and rheumatic diseases.

**What Is Relevant?**

Altered antibody glycosylation may contribute to the pathophysiology of placenta-related complications.
These alterations warrant further investigation to clarify underlying inflammatory and vascular mechanisms.

**Clinical/Pathophysiological Implications?**

Immunoglobulin glycosylation profiling may enable early identification of women at risk for placenta-related complications and offer new targets for personalized prevention and therapy.

Placenta-related pregnancy complications remain a major burden in obstetric care. These conditions, including pregnancy-induced hypertension, preeclampsia, preterm birth, and fetal growth restriction, are associated with substantial maternal and neonatal morbidity and mortality.^1–3^ Despite advances in prenatal care, effective preventive and therapeutic options remain limited, underscoring the need for a deeper understanding of their pathophysiology.

A hallmark of these disorders is impaired maternal vascular adaptation leading to inadequate placental perfusion. Contributing factors include abnormal spiral artery remodeling, endothelial dysfunction, and increased uterine artery resistance, which is often assessed clinically using uterine artery Doppler velocimetry.^1,4^ However, uterine artery Doppler measurements do not reliably predict all cases of placenta-related complications, suggesting that additional, yet uncharacterized, mechanisms may contribute to the development of placenta-related pregnancy complications.

One such mechanism may involve dysregulation of the maternal immune system, which is increasingly recognized as a central contributor to placental pathology.^5^ Ig (immunoglobulins), key components of the adaptive immune system, modulate immune responses during pregnancy. Their function is regulated by glycosylation, the enzymatic attachment of glycans, which influences their structural conformation, receptor binding affinity, and effector functions (Figure 1).^6^ Specific glycan modifications influence immunoglobulin interactions with FcγRs (fragment crystallizable region gamma receptors) and complement components, shaping downstream immune responses.^9^ For example, afucosylation in combination with galactosylation enhances proinflammatory pathways, such as antibody-dependent cellular cytotoxicity via increased affinity for activating FcγRs (eg, FcγRIIIa) on natural killer cells, macrophages, and neutrophils.^9,10^ Furthermore, the absence of galactoses (agalactosylated IgG) is associated with more severe inflammatory disease, because low total IgG galactosylation may shift the immune system toward easier activation by autoantibodies.^11^ In contrast, sialylation of IgG promotes anti-inflammatory signaling through increasing affinity for receptors, such as DC-SIGN (Dendritic Cell-Specific Intercellular adhesion molecule-3-Grabbing Non-integrin) and inhibitory FcγRIIb on macrophages.^9,12,13^

**Figure 1.**
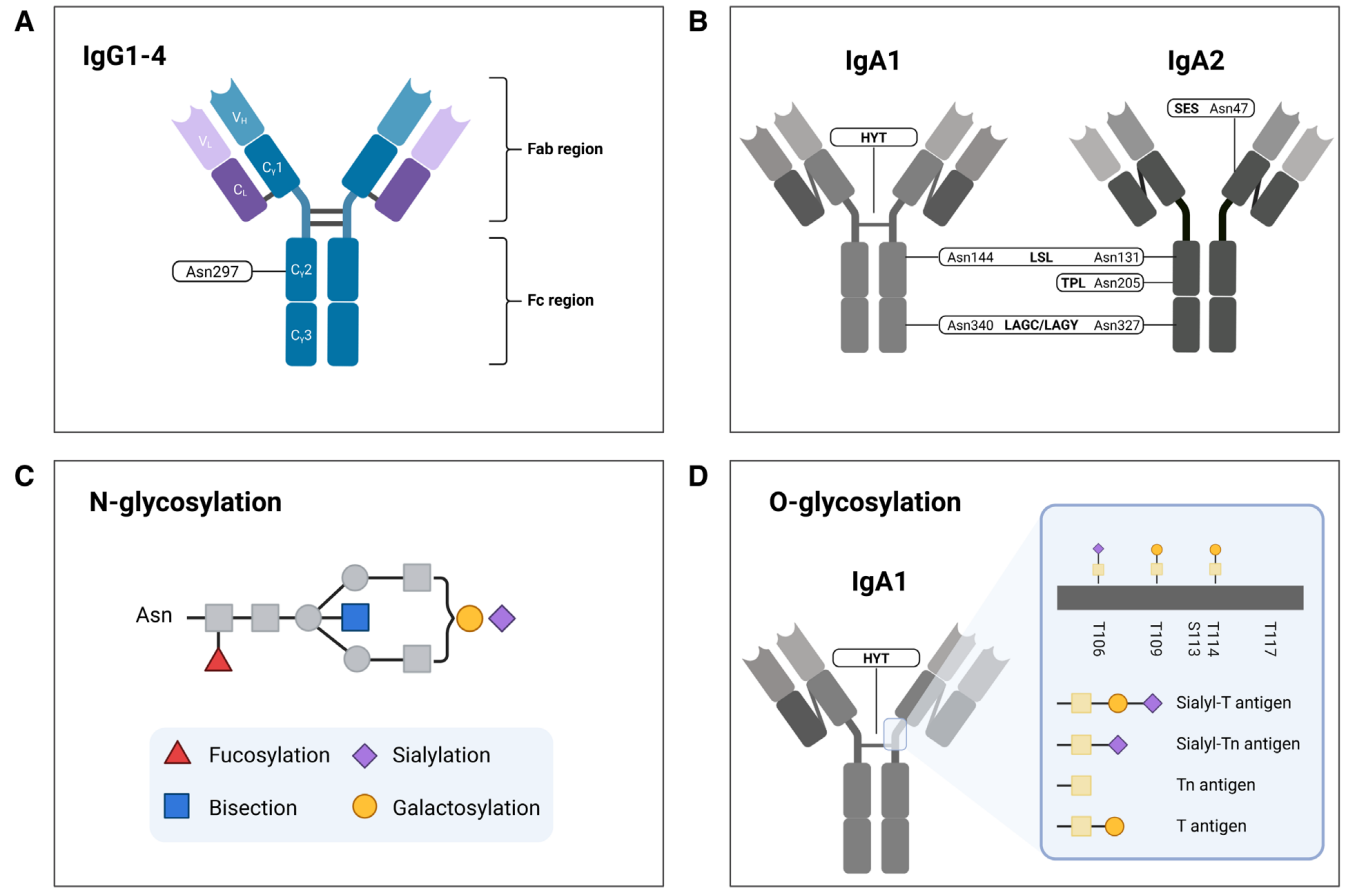
**Overview of IgA (immunoglobulin A) and IgG (immunoglobulin G) glycosylation sites and structures. A**, Schematic IgG molecule showing the N-glycosylation site^6,7^. **B**, Schematic IgA1 and IgA2 molecules showing the N-glycosylation sites and the O-glycosylation region HYT.^8^ IIgA glycopeptides were named according to the first three amino acids of the tryptic peptide sequence: LSL (Leu-Ser-Leu; N-glycosylation at Asn144/Asn131 on IgA1/IgA2), TPL (Thr-Pro-Leu; Asn205 on IgA2), LAG (Leu-Ala-Gly; Asn340/Asn327 on IgA1/IgA2) detected either with a terminal tyrosine (LAGY) or as the truncated form (LAGC), and ENI (Glu-Asn-Ile; Asn71 on the IgA2 J-chain). **C**, Example of a complex biantennary N-glycan structure with glycosylation traits (fucosylation, galactosylation, sialylation, bisection). **D**, Expanded view of the IgA1 hinge region (HYT peptide) illustrating O-glycan heterogeneity.

During pregnancy, IgG (immunoglobulin G) and IgA (immunoglobulin A) glycosylation typically shift toward an anti-inflammatory phenotype, with increased galactosylation and sialylation and reduced bisecting N-acetylglucosamine.^14,15^ These changes occur already within the first trimester and coincide with critical developmental processes, such as trophoblast invasion and placentation, which are also highly sensitive to immune regulation.^16,17^ Disruption of this immunologic balance may, therefore, contribute to the pathogenesis of placenta-related complications.^5,18^ Supporting this, our previous analyses showed that maternal characteristics, including elevated body mass index, primigravidity, and conception via assisted reproductive technologies, were associated with distinct immunoglobulin glycosylation patterns across pregnancy (unpublished work). Notably, the same characteristics are known risk factors for placental disorders.^19,20^

Moreover, altered immunoglobulin glycosylation has been described in autoimmune and cardiovascular diseases, conditions that both share inflammatory and endothelial dysfunction pathways with placental disorders and are associated with increased risk of placental complications.^7,21,22^ These observations indicate that aberrant glycosylation may underlie both immune and vascular maladaptation, representing complementary pathways of interest in the pathogenesis of placental complications.

We hypothesized that pregnancies complicated by placenta-related disorders would exhibit less anti-inflammatory glycosylation profiles, particularly during the first trimester, when placentation occurs. The objective of this study was to characterize longitudinal trajectories of IgG and IgA glycosylation throughout gestation and to determine whether these trajectories differ between pregnancies with and without placenta-related complications.

## Methods

### Data Availability

The data that support the findings of this study are available from the corresponding author on reasonable request.

### Study Design

This study was conducted within the Virtual Placenta Study, an ongoing prospective subcohort embedded in the Rotterdam Periconception Cohort (Predict Study), at the Erasmus MC, University Medical Center, a tertiary care hospital in Rotterdam, the Netherlands.^23,24^ Women aged 18 years or older with a singleton pregnancy were included before 10 weeks of gestational age (GA) between 2017 and 2018. In case of multiple participation, only the first pregnancy per participant was included. Pregnancies ending in miscarriage, with congenital anomalies, or delivered before 24 weeks were excluded. The study was approved by the local ethics committee (MEC-2004-227) and conducted in accordance with the principles of the Declaration of Helsinki. All participants provided written informed consent.

### Clinical Data

At enrollment, participants completed a questionnaire and underwent anthropometric measurements. Pregnancy outcomes were collected from medical records and validated with patient questionnaires. For the primary outcome, pregnancies were classified into 2 groups: those with placenta-related complications and those without. Placenta complications included pregnancy-induced hypertension, preeclampsia, preterm birth (<37 weeks GA), and small-for-GA (SGA). Pregnancy-induced hypertension was defined as new-onset hypertension (systolic ≥140 mm Hg and diastolic ≥90 mm Hg) after 20 weeks GA. Preeclampsia was defined as hypertension with proteinuria, fetal growth restriction, and maternal organ damage.^25^ Small-for-GA was defined as a birthweight percentile below the 10th percentile compared with a reference curve.^26^

### Glycosylation Measurements

Maternal blood was collected at 9, 11, 13, 22, and 32 weeks GA at our outpatient clinic and directly transported to the lab. Neonatal cord blood was collected immediately after birth. Some cord blood samples were collected from women who delivered at other hospitals or at home births. In such cases, transport delays were unavoidable. These cord blood samples were collected in vacutainers, stored at room temperature, and sent by post as soon as possible, but always within 48 hours after collection. Samples were allowed to clot for ≥60 minutes at room temperature, centrifuged at 2000×*g* for 10 minutes at 4 °C, aliquoted (4×0.5 mL), and stored at −80 °C. IgG and IgA glycosylation were profiled at the glycopeptide level using a validated protocol.^27^ In short, immunoglobulins were affinity-captured, reduced, alkylated, and digested with trypsin, followed by nanoLC–MS. Raw data were processed and quality-controlled using LaCyTools software, and glycosylation traits (eg, fucosylation, galactosylation, sialylation, bisection) were derived from normalized glycoform abundances (Table S1) and reported per glycosylation site (Figure 1).^28^ Laboratory staff performing LC–MS analyses were blinded to clinical data.

### Statistical Analysis

Primary outcomes included glycosylation trajectories in pregnancies with and without placenta-related complications. For each Z-standardized glycan trait, we fitted linear mixed-effects models with a natural cubic spline for GA (2 df) and its interaction with complication status, including a random intercept for participant. Adjusted models included body mass index, primigravidae status, and mode of conception (natural versus in vitro fertilization/intracytoplasmic sperm injection) as covariates, based on prior findings in our cohort (unpublished work). Maternal age was not included, as these prior analyses showed no association with glycosylation traits, and the limited number of complications required a parsimonious model. Mode of conception was classified as natural (spontaneous conception, ovulation induction, or intrauterine insemination) or in vitro fertilization with or without intracytoplasmic sperm injection. Estimated marginal means were obtained at 9, 11, 13, 22, and 32 weeks; between-group contrasts (yes–no) with 95% CIs were summarized at 11, 22, and 32 weeks. False discovery rate (FDR) correction was applied across traits and time points, and a *q* <0.05 was considered statistically significant. Sensitivity analyses included regrouping of women with spontaneous preterm birth but without any other placenta-related complications as not having a placenta-related complication. A second analysis compared pregnancies with hypertensive disorders (pregnancy-induced hypertension or preeclampsia) to those without complications, excluding isolated SGA or preterm births. Cord blood IgG and IgA glycosylation traits were standardized (*Z* scores) and analyzed cross-sectionally. Associations with pregnancy outcomes were assessed using Spearman correlations, with calculation of coefficients (r) and corresponding *P* values.

## Results

A total of 193 ongoing pregnancies were included. Of these, 54 were complicated by at least 1 placenta-related disorder: preterm birth occurred in 44% (n=24), preeclampsia in 13% (n=7), gestational hypertension in 28% (n=15), and SGA in 44% (n=24; Table). Co-occurrence of complications is summarized in Table S2. The remaining 139 pregnancies were not affected by any of these complications. Mean maternal age at conception was 32 years and was higher in uncomplicated than in complicated pregnancies (32.6 versus 31.1 years; *P*=0.033). Baseline characteristics were otherwise comparable (Table).

**Table.**
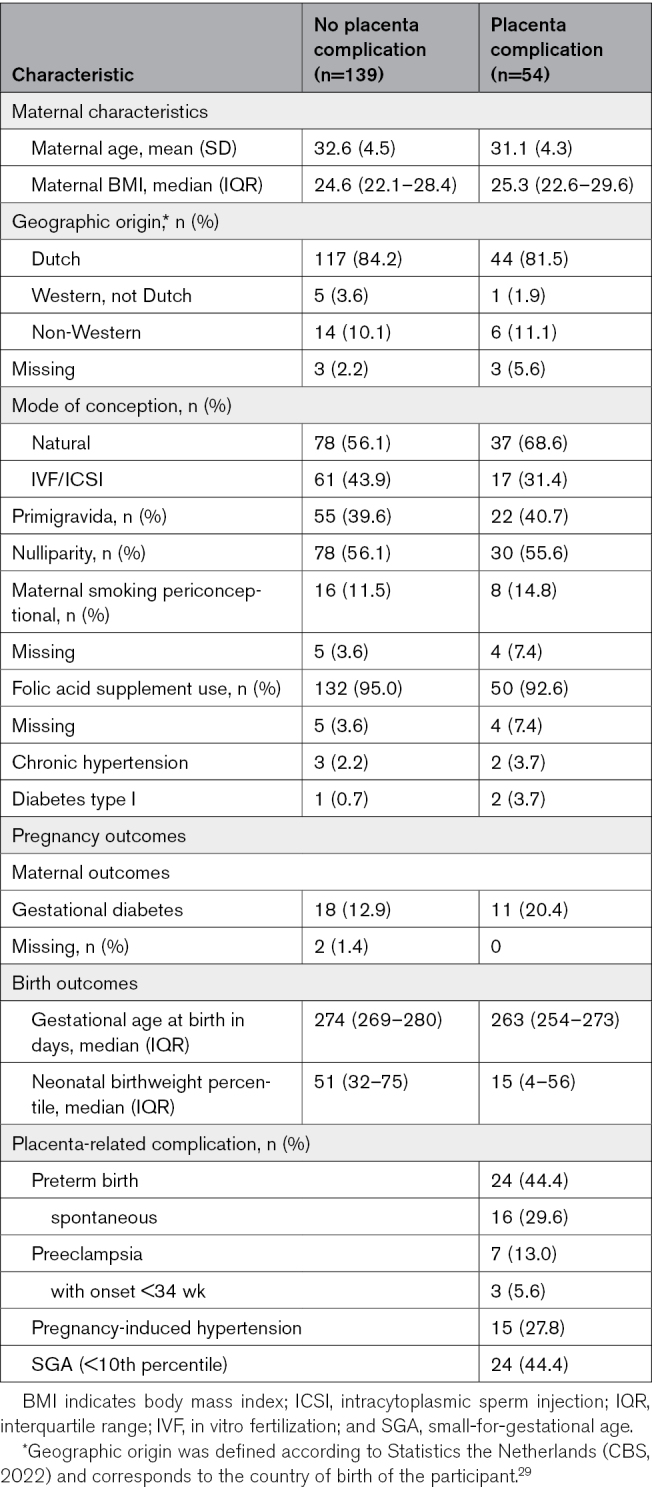
Baseline Table Stratified by Placenta-Related Complications

### Glycosylation Trajectories

**Figure 2.**
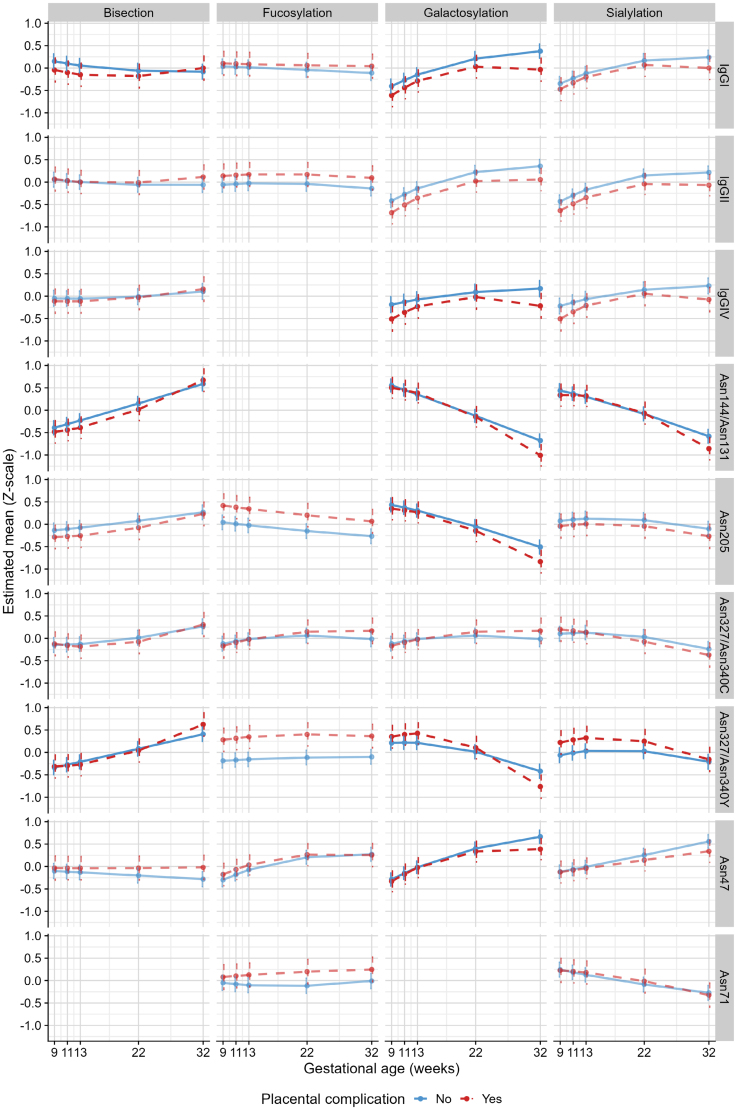
**Trajectories of maternal IgG (immunoglobulin G) and IgA (immunoglobulin A) glycosylation traits across gestation.** Lines represent estimated marginal means (Z-standardized values) for pregnancies with placenta-related complications (red, dashed) and without complications (blue, solid), derived from linear mixed-effects models. Each column facet represents a glycosylation trait (fucosylation, bisection, galactosylation, or sialylation), and each row facet represents an individual glycosylation site. Error bars indicate 95% CIs of the estimated marginal means. More intense line colors denote traits with a statistically significant interaction term (placenta complication × gestational age) in the mixed model, while lighter colors indicate nonsignificant interactions.

In the crude models, when comparing glycosylation trajectories across pregnancy between placenta-complications and uncomplicated pregnancies, several differences were observed (Figure 2).

For bisection, a significant interaction term was observed for Asn327/Asn340Y, Asn144/ASN131, and IgG1 (Figure 2). For Asn144/Asn131 and IgGI, bisection is lower in the first and second trimester, and on all 3 sites, bisection increased more strongly towards the third trimester in pregnancies complicated by placenta-related disorders.

For fucosylation, no glycosylation sites showed a significant interaction between GA and complication status, indicating that the overall trajectory across pregnancy was similar between women with and without placenta-related complications. However, mean fucosylation levels were consistently higher in the complication group across all measured sites. This difference reached statistical significance at 2 IgA sites: Asn327/Asn340Y (mean *Z* score difference across pregnancy: 0.45–0.52; *q*=0.002–0.004) and Asn205 (mean *Z* score difference: 0.33–0.37; *q*=0.038).

A trend towards lower galactosylation throughout pregnancy was observed in women with placenta-related complications across all IgG subtypes, with significant interaction with GA (*q*=0.021–0.025) for IgG1 and IgGIV (Figure 2). At IgA sites Asn144/Asn131, Asn205, and Asn327/Asn340Y, galactosylation decreased more strongly towards the third trimester in the complication group (*q*=0.000–0.024). In contrast, galactosylation on Asn327/Asn340C and Asn47 increased over pregnancy in both groups, but significantly less at the Asn47 site for placenta complications (*q*=0.021; Figure 2).

For sialylation, significant trajectory differences were found at Asn144/Asn131A and Asn327/Asn340Y (*q*=0.021–0.026). Women with placenta-related complications showed higher sialylation at Asn327/Asn340Y in the first trimester, and a steeper decline on ASN144/ASN131 and Asn327/Asn340Y after the second trimester (*q*=0.026). On the other sites, sialylation was generally lower in the complication group, either consistently across pregnancy (IgG I–IV, Asn205), with a stronger decline (Asn327/Asn340C), or with a more modest increase (Asn47) towards the third trimester (Figure 2).

#### Adjusted Models

In the models adjusted for maternal body mass index, primigravidae status, and mode of conception, trajectories were similar to the unadjusted analyses, with the same interaction terms remaining significant. Local contrasts from the adjusted and unadjusted models were highly consistent in both effect size and direction. The only exception was Asn144/Asn131 galactosylation at 32 weeks’ GA, which became significant after adjustment (*Z* score difference, −0.35; *q*=0.041).

#### O-glycosylation (IgA1)

**Figure 3.**
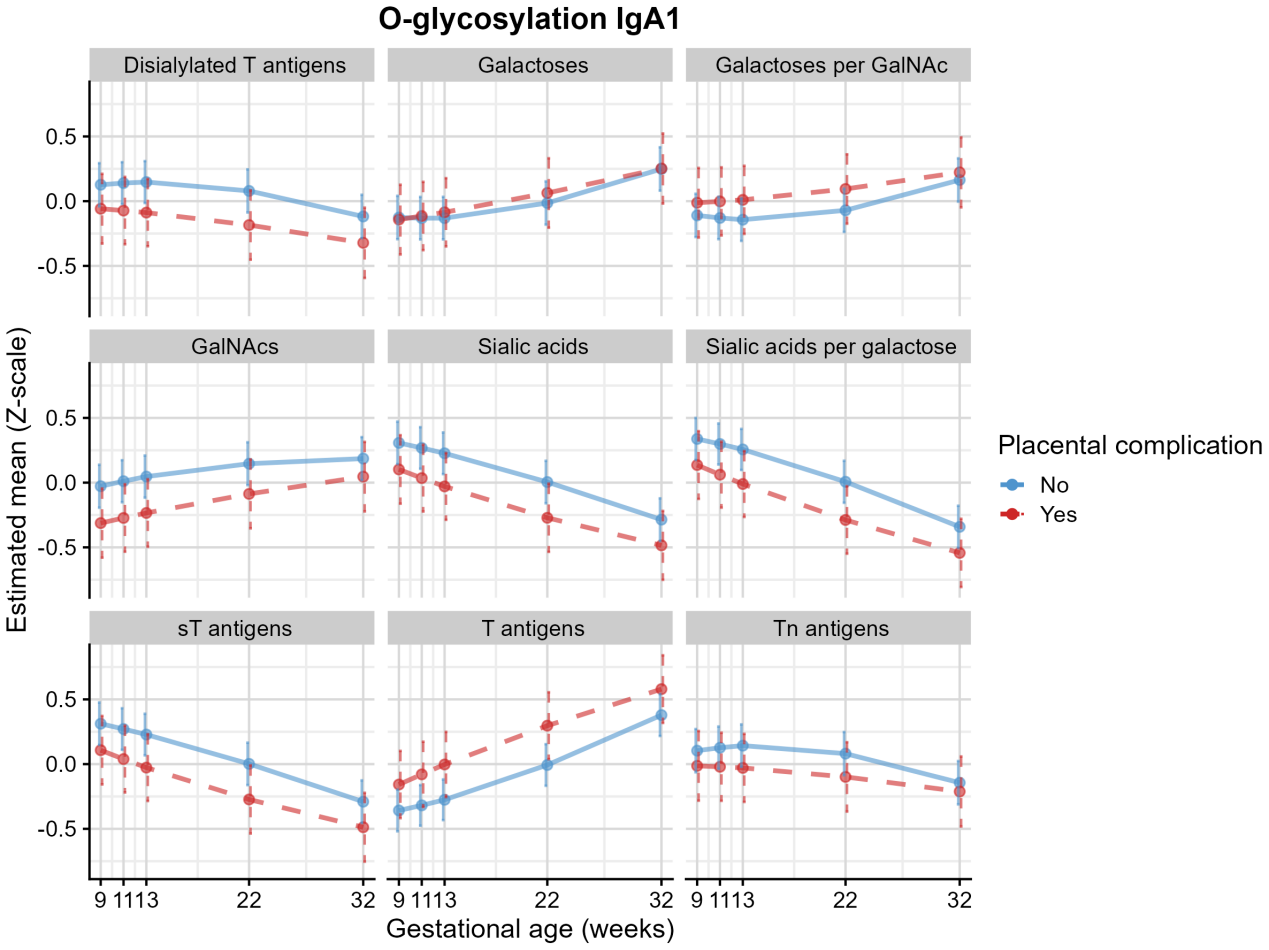
**Estimated trajectories of IgA1 (immunoglobulin A) O-glycosylation (HYT region) across gestation in pregnancies with and without placenta-related complications.** Lines represent estimated marginal means (Z-standardized values) for women with placenta-related complications (red, dashed) and uncomplicated pregnancies (blue, solid), derived from linear mixed-effects models. Each column facet corresponds to an O-glycosylation feature (eg, sialic acids, N-Acetylgalactosamine (GalNAcs), T-antigens, sT-antigens, or disialylated T-antigens). Error bars denote 95% CIs of the estimated marginal means.

None of the O-glycosylation traits measured at the HYT region (IgA1) showed significantly different trajectories between pregnancies with and without placenta-related complications, either before or after FDR adjustment (Figure 3). Nevertheless, a consistent trend was observed in the complication group, characterized by lower sialylation across pregnancy. This was reflected by reduced disialylated T antigens (mean *Z* score difference –0.20 to −0.26; *q*=0.210), lower levels of total sialic acids and sialic acids per galactose (mean difference between −0.20 and −0.29), and reduced sT antigens (−0.20 to −0.27). In addition, N-Acetylgalactosamine levels were lower (−0.14 to −0.28), while T antigens were relatively higher (0.20–0.30).

### Cord Blood

**Figure 4.**
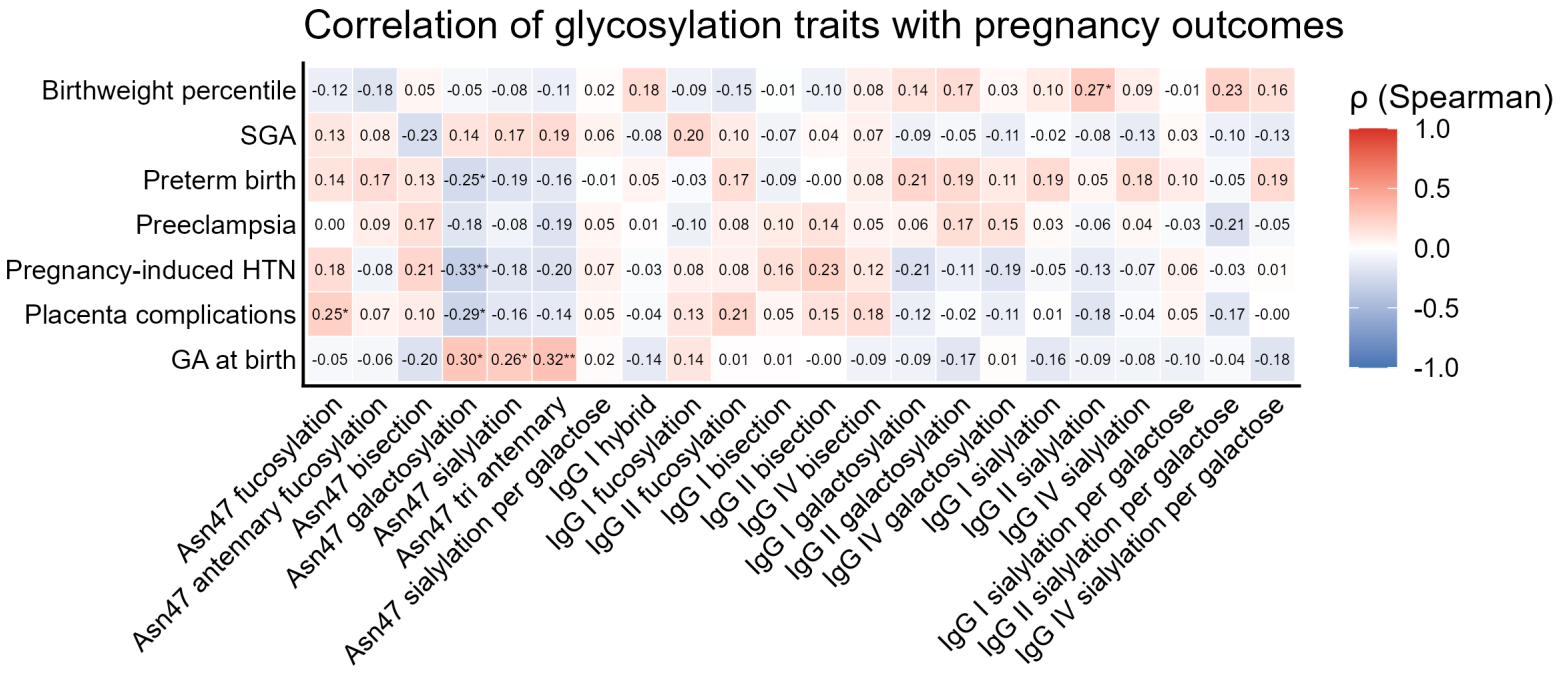
**Correlation matrix of glycosylation traits measured in neonatal cord blood (n=75) with clinical pregnancy outcomes.** GA indicates gestational age; HTN, hypertension; IgG, immunoglobulin G; and SGA, small-for-gestational-age. Significance levels: *P<0.05; **P<0.01.

Correlation analyses revealed modest but consistent associations between cord blood glycosylation and pregnancy outcomes. Placenta-related complications were negatively correlated with galactosylation and sialylation, and positively correlated with fucosylation and bisection in cord blood (Figure 4).

Among IgG traits, IgG2 sialylation showed the strongest positive correlation with birthweight percentile (*r*=0.27; *P*<0.05). More generally, both sialylation and galactosylation tended to correlate positively with birthweight percentile and negatively with SGA. In contrast, IgG bisection displayed positive correlations with placenta-related complications, particularly pregnancy induced hypertension and preeclampsia, with the highest coefficients observed for IgG2. Interestingly, galactosylation showed a mixed pattern: it correlated positively with preeclampsia (*r*=0.06–0.17) and preterm birth (*r*=0.11–0.21), but negatively with gestational hypertension and SGA (*r*≈–0.05 to –0.20).

For IgA glycosylation, only the Asn47 site (IgA2) could be measured in cord blood. Significant positive correlations with GA at birth were observed for more complex Asn47 glycosylation traits (sialylation, galactosylation, and tri-antennary structures; *r*=0.26–0.32). In addition, Asn47 galactosylation correlated negatively with overall placenta-related complications (*r*=–0.29; *P*<0.05), preterm birth (*r*=−0.25), and gestational hypertension (*r*=–0.33; *P*<0.01).

### Sensitivity Analyses

In a sensitivity analysis reclassifying women with spontaneous preterm birth (n=11) as pregnancies without complications, fewer significant interactions were observed. However, effect directions were comparable to the main analysis (Figure S1). The remaining significant interactions after FDR correction included galactosylation of IgGI, IgG4, and Asn327/Asn340Y, IgGI bisection, and Asn47 tri-antennary structures. Glycosylation traits with significant mean differences per trimester after FDR correction were unchanged. When comparing women with hypertensive complications to those without any placental complications, effect directions were largely consistent with the main analysis, although fewer associations reached statistical significance (Figure S2). Across all IgG subclasses, galactosylation and sialylation remained lower in hypertensive pregnancies, with the pronounced decline in Asn327/Asn340Y and Asn205 galactosylation towards late gestation. In the third trimester, IgG1 galactosylation was significantly reduced in hypertensive pregnancies (−0.58 *Z* score; q=0.033), as was Asn205 galactosylation. Asn327/Asn340Y fucosylation was higher throughout pregnancy in the hypertensive group (0.57–0.63 *Z* score). Interestingly, the number of N-Acetylgalactosamines on the HYT region (O-glycosylation) was significantly lower in hypertensive disorders in all trimesters (−0.64 to −0.51 *Z* score difference; [*q*=0.011–0.026]; Figure S3).

## Discussion

### Key Findings

Specifically, women with complications exhibited higher fucosylation across IgG and IgA sites and lower galactosylation across all IgG subtypes throughout gestation. IgA galactosylation declined more steeply toward the third trimester, while sialylation was consistently lower in the complication group—either across the entire pregnancy (IgG I–IV, Asn205) or with a more pronounced decline towards the third trimester (Asn144/Asn131 and Asn327/Asn340Y). Bisection showed less pronounced differences but tended to be lower for Asn144/Asn131 and IgG1 in early pregnancy, increasing more strongly toward the third trimester at several sites in complicated pregnancies. None of the O-glycosylation traits at IgA1 remained significant after FDR correction, although trends toward lower sialylation persisted. In cord blood, placenta-related complications were similarly associated with lower galactosylation and sialylation, and higher fucosylation and bisection across both IgG and IgA traits, reflecting the observed proinflammatory shift at delivery.

### Interpretation of Findings

#### Immunologic Effects

During a healthy pregnancy, maternal immunity adapts to promote fetal tolerance while maintaining host defense. This adaptation is reflected in IgG glycosylation, which gradually shifts toward an anti-inflammatory profile with increasing galactosylation and sialylation of the fragment crystallizable region.^15^ In pregnancies complicated by preeclampsia, fetal growth restriction, or preterm birth, this anti-inflammatory shift appears attenuated: Women with placenta-related complications consistently showed lower IgG galactosylation and sialylation, together with higher fucosylation.

These glycosylation changes might reflect the underlying immune dysregulation in these disorders, which are characterized by a proinflammatory maternal–fetal interface, dominated by M1-like decidual macrophages, increased proinflammatory cytokine production (eg, TNF-α [Tumor necrosis factor-alpha], IL-1β [Interleukin-1 beta], IL-6 [Interleukin-6]), and complement overactivation.^18,30,31^ Notably, a similar proinflammatory IgG glycosylation profile has been described in autoimmune and autoinflammatory conditions such as systemic lupus erythematosus and IgA nephropathy—both of which are associated with increased risk of placental complications and share clinical and pathophysiological features with preeclampsia.^32–34^ Together, these observations suggest that altered glycosylation may play a mechanistic role in placenta complications. Previous studies have indeed observed altered glycosylation of trophoblast cells and placental tissue of preeclampsia patients.^35^ Bueno-Sanchez et al^35^ describe more proinflammatory glycosylation changes in these placental cells, which were associated with modulation of maternal innate immune responses, including natural killer cell activation. However, these studies did not assess immunoglobulin glycosylation, which is easier to measure and was evaluated longitudinally in our study, providing additional insight into immune alterations during pregnancy. This distinction highlights the novel contribution of our work. Sialylated IgG, for example, generally exerts anti-inflammatory effects by reducing affinity for activating FcγRs and enhancing engagement of inhibitory receptors such as FcγRIIb and Siglec (Sialic acid-binding immunoglobulin-like lectin) 7.^17,36,37^ In macrophages, these interactions promote M2-like polarization and suppress inflammatory signaling via DC-SIGN and other lectins.^12,17^ Reduced sialylation may impair these regulatory mechanisms, limiting anti-inflammatory receptor engagement and lowering the threshold for macrophage activation, thereby shifting the decidual immune environment toward inflammation.

Increased IgG1 galactosylation enhances C1q binding and facilitates controlled activation of the classical complement pathway, supporting physiological immune and vascular adaptation during pregnancy.^37–40^ In addition, galactosylated IgG can inhibit C5a-mediated neutrophil activation through cooperative engagement of FcγRIIB and Dectin-1, suggesting that lower galactosylation may remove an important brake on inflammatory effector functions.^38^ We therefore hypothesize that loss of IgG galactosylation contributes to the pathophysiology of placenta-related pregnancy complications through dysregulated complement-mediated vascular injury.

We also identified higher IgG fucosylation in complicated pregnancies. Lower fucosylation is known to enhance antibody-dependent cellular cytotoxicity via FcγRIIIa,^10^ and elevated fucosylation may attenuate this activating immune response, potentially contributing to immune modulation in these cases. However, the association between fucosylation and inflammatory diseases can differ: a lower fucosylation is observed in women with systemic lupus erythematosus and ulcerative colitis, while in Crohn disease, IgG fucosylation is higher compared with healthy controls.^22,41^ Moreover, in rheumatoid arthritis, elevated IgG1 fucosylation has been associated with greater disease activity during pregnancy, suggesting a potential link to systemic inflammation.^42^ Thus, the potential mechanisms by which increased fucosylation might contribute to placental dysfunction remain to be elucidated.

IgA also showed different glycosylation profiles in pregnancies complicated by preeclampsia, SGA, or preterm birth, with lower galactosylation and sialylation, especially towards the third trimester for Asn144/Asn131, Asn327/340Y, and Asn205 (IgA2 only), and increased fucosylation throughout pregnancy for Asn327/Asn340Y and Asn205. How these changes may contribute to placenta-related complications is less clear, as IgA’s role in proinflammatory pathways is less well characterized than that of IgG. IgA can engage FcαRI (CD89) on monocytes, macrophages, and neutrophils, triggering inflammatory responses including cytokine release and phagocytosis.^43^ Although the influence of IgA glycosylation on FcαRI signaling is not well understood, reduced sialylation may hypothetically lower the threshold for immune activation.

Interestingly, in a sensitivity analysis focusing solely on hypertensive disorders, we observed significantly higher N-Acetylgalactosamines on the HYT region of IgA1. Altered N-Acetylgalactosamine glycosylation in this region has been implicated in immune complex formation and vascular inflammation, in IgA nephropathy and IgA vasculitis.^44^ Patients with IgA nephropathy also have higher risks of preeclampsia, suggesting a potential mechanistic link to hypertensive pathology.^32^ In systemic lupus erythematosus, a decrease in galactosylation in this region has been observed,^43^ but this was not seen in our sensitivity analysis.

In rheumatoid arthritis pregnancies, IgA glycosylation changes in pregnancy were not associated with disease activity, suggesting a smaller role in modulating systemic inflammation compared with IgG.^45^ This may in part reflect IgA’s lower abundance in serum (≈15% of total immunoglobulin). Although no studies have yet linked IgA glycosylation directly to placental dysfunction, its potential role in local immune activation at the maternal–fetal interface warrants further investigation.

#### Vascular Effects

Beyond immune cell modulation, glycosylation may also influence vascular tone. In models of obesity-induced hypertension, hyposialylated IgG induced endothelial dysfunction via FcγRIIB-dependent inhibition of eNOS (endothelial nitric oxide synthase), impairing nitric oxide–mediated vasodilation.^46^ As impaired eNOS signaling is implicated in preeclampsia and gestational hypertension, a similar mechanism may be relevant in pregnancy.^47^ This is further supported by epidemiological evidence linking low IgG galactosylation and sialylation to increased cardiovascular risk and endothelial dysfunction in nonpregnant women.^7,48,49^

#### Cord Blood

Because neonatal IgG is transferred across the placenta, the observed negative correlations between placenta-related disorders and IgG galactosylation and sialylation likely reflect altered maternal glycosylation profiles rather than selective placental transfer, which we could not differentiate due to the absence of maternal samples at delivery. Interestingly, galactosylation showed a mixed pattern: it correlated positively with preeclampsia and preterm birth, but negatively with gestational hypertension and small-for-gestational-age birth. These divergent associations may indicate distinct underlying mechanisms or reflect differences in disease severity. The observed higher galactosylation in our study contrasts with previous work by Twisselman et al,^50^ who reported lower IgG galactosylation and sialylation in infants born before 28 weeks GA, reflecting a more proinflammatory profile. In their study, maternal IgG glycosylation was measured only at birth, without longitudinal sampling, and did not show significant differences by GA at delivery. Additionally, adverse pregnancy outcomes were not considered in their analysis. Our study, however, uses a methodology that allows us to assess glycosylation changes per subclass, which provides a more detailed picture of maternal immune alterations over time.

Nevertheless, as we could not adjust for potential confounding factors, these findings should be interpreted cautiously, and further studies are needed to disentangle the direction and causality of these relationships. Asn47 glycosylation traits in IgA2 also correlated with GA, likely reflecting the fetal developmental stage, as IgA is produced endogenously by the fetus.

#### Strengths and Limitations

This study’s longitudinal design allowed characterization of glycosylation trajectories from the first trimester onwards and to investigate them in relation to placenta-related complications. Sensitivity analyses supported the robustness of our findings. Reclassifying spontaneous preterm births did not substantially alter the overall pattern of associations, though significance was attenuated, likely due to reduced statistical power. Notably, 5 of 16 spontaneous preterm births co-occurred with SGA, suggesting shared pathophysiology and supporting their inclusion in the complication group. In hypertensive disorders of pregnancy, consistent differences in IgG galactosylation and sialylation were observed, though the limited sample size reduced power for subtler effects. Nonetheless, several limitations should be acknowledged. Although we hypothesize that glycosylation changes may precede and contribute to the development of placenta-related complications, the observational nature of this study prevents us from establishing causality, and it remains possible that these changes are a consequence rather than a cause of the complications. Although we adjusted for known confounders such as body mass index, mode of conception, and parity, residual confounding from unmeasured factors cannot be excluded, inherent to the study design. As this was an exploratory study with sample size constrained by longitudinal sample availability, we did not conduct a priori power calculations. The sample size, while sufficient for initial exploration, was modest and may have limited statistical power, particularly in subgroup analyses such as those restricted to hypertensive disorders. Furthermore, while FDR correction was applied to account for multiple testing, this conservative approach may have masked biologically relevant associations. To address this, we also examined trends across immunoglobulin subtypes and glycosylation traits, which showed consistency of our findings beyond strict statistical thresholds. This internal consistency supports the robustness of the overall pattern, while recognizing that effect size estimates should be confirmed in independent cohorts.

### Perspectives

Maternal IgA and IgG glycosylation profiles are altered in placenta-related complications. These changes may not only mirror immune dysregulation toward a proinflammatory state but also contribute functionally to disease pathogenesis through enhanced Fcγ receptor and complement-mediated inflammation. Glycosylation thus represents a promising integrative biomarker linking maternal immune status to placental health.

To determine whether these alterations are a cause or consequence of disease, future research should aim to disentangle temporal and mechanistic relationships. Experimental approaches such as glycoengineering and in vitro placental models may help clarify causal pathways. In addition, combining animal models of preeclampsia with human immunoglobulin glycosylation profiling may help clarify underlying mechanisms. In particular, IgG glycosylation warrants further investigation due to its abundance, established associations with disease activity in pregnancy, and links to long-term cardiovascular risk.^7,42^ Experimental evidence supports the vascular relevance of IgG sialylation: supplementation with N-acetyl-D-mannosamine restored IgG sialylation and prevented hypertension in mice, suggesting therapeutic potential through modulation of vascular inflammation and endothelial nitric oxide synthase signaling.^46^ These findings warrant further exploration of N-acetyl-D-mannosamine supplementation in human pregnancy, particularly to assess whether it enhances IgG sialylation and how this, in turn, may influence blood pressure.

Within IgA, the Asn327/Asn340Y and Asn144/Asn131glycopeptides emerged as promising biomarker candidates, showing consistent differences across multiple glycosylation traits and subtypes. The pronounced third-trimester Asn144/Asn131 differences may, however, reflect downstream consequences rather than causal involvement. Ultimately, elucidating the mechanistic and temporal dynamics of maternal immunoglobulin glycosylation may enable early risk prediction, personalized prevention, and novel therapeutic approaches to improve maternal and fetal outcomes.

## Article Information

### Disclosures

None.

### Supplemental Material

Tables S1 and S2

Figures S1–S3

## Supplementary Material


