# Supplementary material for: Longitudinal Maternal IgG and IgA Glycosylation Profiles in Pregnancy Reveal Early Immune Alterations in Placenta-Related Complications: The Rotterdam Periconception Cohort
Source: Hypertension. 2026 Jun 26;83(9):e26531. doi: 10.1161/HYPERTENSIONAHA.126.26531 (PMC13480331; doi:10.1161/HYPERTENSIONAHA.126.26531)
Supplement: Supplementary file 1 [file hyp-83-e26531-s001.pdf]

Online Supplemental Material

Title: Longitudinal Maternal IgG and IgA Glycosylation Profiles in Pregnancy Reveal Early Immune Alterations in Placenta-related Complications: The Rotterdam Periconception Cohort

Authors: Lotte W. Voskamp[1,2], Melek Rousian[1], Anna Daniels[1], Manfred Wuhrer[3], A.H. Jan Danser[2], Régine P.M. Steegers-Theunissen[1], Koen Verdonk[2]

Affiliations:

1. Department of Obstetrics and Gynecology, Erasmus MC University Medical Center, Rotterdam, the Netherlands
2. Department of Internal Medicine, Erasmus University Medical Center, Rotterdam, The Netherlands.
3. Center for Proteomics and Metabolomics, Leiden University Medical center, Leiden, The Netherlands.

Running/Long title: IgG and IgA Glycosylation Profiles in Placenta-Related Pregnancy Complications

Short title: IgG and IgA Glycosylation in Pregnancy Complications

Corresponding author:

Name: dr. Koen Verdonk

Institution: Erasmus University Medical Center, Rotterdam, the Netherlands

E-mail: k.verdonk@erasmusmc.nl Phone: +31634199289

Supplemental Tables

Table S1: Calculation formulas of composite glycosylation traits. LSL = Asn144/Asn131, SES = Asn47, TPL = Asn205, ENI = Asn71, LAGY = Asn327/Asn340Y, LAGC = Asn327/Asn340C.

Glycosylation trait	Formula
IgGI_fucosylation	(1 * (IgGI1H3N4F1 + IgGI1H4N4F1 + IgGI1H5N4F1 + IgGI1H4N4F1S1 + IgGI1H5N4F1S1 + IgGI1H5N4F1S2 + IgGI1H3N5F1 + IgGI1H4N5F1 + IgGI1H5N5F1 + IgGI1H4N5F1S1 + IgGI1H5N5F1S1 + IgGI1H4N3F1)) / (IgGI1H3N4F1 + IgGI1H4N4F1 + IgGI1H5N4F1 + IgGI1H4N4F1S1 + IgGI1H5N4F1S1 + IgGI1H5N4F1S2 + IgGI1H3N5F1 + IgGI1H4N5F1 + IgGI1H5N5F1 + IgGI1H4N5F1S1 + IgGI1H5N5F1S1 + IgGI1H3N4 + IgGI1H4N4 + IgGI1H5N4 + IgGI1H5N4S1 + IgGI1H3N5 + IgGI1H4N5 + IgGI1H5N5 + IgGI1H5N5S1 + IgGI1H4N3F1) * 100
IgGI_bisection	(1 * (IgGI1H3N5F1 + IgGI1H4N5F1 + IgGI1H5N5F1 + IgGI1H4N5F1S1 + IgGI1H5N5F1S1 + IgGI1H3N5 + IgGI1H4N5 + IgGI1H5N5 + IgGI1H5N5S1)) / (IgGI1H3N4F1 + IgGI1H4N4F1 + IgGI1H5N4F1 + IgGI1H4N4F1S1 + IgGI1H5N4F1S1 + IgGI1H5N4F1S2 + IgGI1H3N5F1 + IgGI1H4N5F1 + IgGI1H5N5F1 + IgGI1H4N5F1S1 + IgGI1H5N5F1S1 + IgGI1H3N4 + IgGI1H4N4 + IgGI1H5N4 + IgGI1H5N4S1 + IgGI1H3N5 + IgGI1H4N5 + IgGI1H5N5 + IgGI1H5N5S1 + IgGI1H4N3F1) * 100
IgGI_galactosylation	(0.5 * (IgGI1H4N4F1 + IgGI1H4N4F1S1 + IgGI1H4N5F1 + IgGI1H4N5F1S1 + IgGI1H4N4 + IgGI1H4N5) + 1 * (IgGI1H5N4F1 + IgGI1H5N4F1S1 + IgGI1H5N4F1S2 + IgGI1H5N5F1 + IgGI1H5N5F1S1 + IgGI1H5N4 + IgGI1H5N4S1 + IgGI1H5N5 + IgGI1H5N5S1 + IgGI1H4N3F1)) / (IgGI1H3N4F1 + IgGI1H4N4F1 + IgGI1H5N4F1 + IgGI1H4N4F1S1 + IgGI1H5N4F1S1 + IgGI1H5N4F1S2 + IgGI1H3N5F1 + IgGI1H4N5F1 + IgGI1H5N5F1 + IgGI1H4N5F1S1 + IgGI1H5N5F1S1 + IgGI1H3N4 + IgGI1H4N4 + IgGI1H5N4 + IgGI1H5N4S1 + IgGI1H3N5 + IgGI1H4N5 + IgGI1H5N5 + IgGI1H5N5S1 + IgGI1H4N3F1) * 100
IgGI_sialylation	(0.5 * (IgGI1H4N4F1S1 + IgGI1H5N4F1S1 + IgGI1H4N5F1S1 + IgGI1H5N5F1S1 + IgGI1H5N4S1 + IgGI1H5N5S1) + 1 *

	IgGI1H5N4F1S2) / (IgGI1H3N4F1 + IgGI1H4N4F1 + IgGI1H5N4F1 + IgGI1H4N4F1S1 + IgGI1H5N4F1S1 + IgGI1H5N4F1S2 + IgGI1H3N5F1 + IgGI1H4N5F1 + IgGI1H5N5F1 + IgGI1H4N5F1S1 + IgGI1H5N5F1S1 + IgGI1H3N4 + IgGI1H4N4 + IgGI1H5N4 + IgGI1H5N4S1 + IgGI1H3N5 + IgGI1H4N5 + IgGI1H5N5 + IgGI1H5N5S1 + IgGI1H4N3F1) * 100
IgGI_hybrid	1 * (IgGI1H6N3F1S1 + IgGI1H6N4F1)
IgGII_fucosylation	1 * (IgGII1H3N4F1 + IgGII1H4N4F1 + IgGII1H5N4F1 + IgGII1H4N4F1S1 + IgGII1H5N4F1S1 + IgGII1H3N5F1 + IgGII1H4N5F1 + IgGII1H5N5F1 + IgGII1H5N5F1S1)
IgGII_bisection	1 * (IgGII1H3N5F1 + IgGII1H4N5F1 + IgGII1H5N5F1 + IgGII1H5N5F1S1)
IgGII_galactosylation	0.5 * (IgGII1H4N4F1 + IgGII1H4N4F1S1 + IgGII1H4N5F1 + IgGII1H4N4) + 1 * (IgGII1H5N4F1 + IgGII1H5N4F1S1 + IgGII1H5N5F1 + IgGII1H5N5F1S1)
IgGII_sialylation	0.5 * (IgGII1H4N4F1S1 + IgGII1H5N4F1S1 + IgGII1H5N5F1S1)
IgGIV_bisection	1 * (IgGIV1H3N5F1 + IgGIV1H4N5F1 + IgGIV1H5N5F1)
IgGIV_galactosylation	0.5 * (IgGIV1H4N4F1 + IgGIV1H4N4F1S1 + IgGIV1H4N5F1) + 1 * (IgGIV1H5N4F1 + IgGIV1H5N4F1S1 + IgGIV1H5N5F1)
IgGIV_sialylation	0.5 * (IgGIV1H4N4F1S1 + IgGIV1H5N4F1S1)
IgGI_sialylation_per_galactose	IgGI_sialylation / IgGI_galactosylation * 100
IgGII_sialylation_per_galactose	IgGII_sialylation / IgGII_galactosylation * 100
IgGIV_sialylation_per_galactose	IgGIV_sialylation / IgGIV_galactosylation * 100
SES_fucosylation	1 * (SES1H4N5F1 + SES1H4N5F1S1 + SES1H4N5F2 + SES1H4N5F2S1 + SES1H5N4F1S2 + SES1H5N5F1S1 + SES1H5N5F1S2 + SES1H5N6F1 + SES1H5N6F2 + SES1H5N6F1S1 + SES1H5N6F1S2 + SES1H5N6F2S2)
SES_antennary_fucosylation	1 * (SES1H4N5F2 + SES1H4N5F2S1 + SES1H5N6F2 + SES1H5N6F2S2)
SES_bisection	1 * (SES1H4N5F1 + SES1H4N5F1S1 + SES1H4N5F2 + SES1H4N5F2S1 + SES1H5N5S1 + SES1H5N5F1S1 + SES1H5N5F1S2 + SES1H5N6F1 + SES1H5N6F2 + SES1H5N6F1S1 + SES1H5N6F1S2 + SES1H5N6F2S2)

SES_galactosylation	0.5 * (SES1H4N5F1 + SES1H4N5F1S1 + SES1H4N5F2 + SES1H4N5F2S1) + 1 * (SES1H5N4F1S2 + SES1H5N5S1 + SES1H5N5F1S1 + SES1H5N5F1S2) + 0.667 * (SES1H5N6F1 + SES1H5N6F2 + SES1H5N6F1S1 + SES1H5N6F1S2 + SES1H5N6F2S2)
SES_sialylation	0.5 * (SES1H4N5F1S1 + SES1H4N5F2S1 + SES1H5N5S1 + SES1H5N5F1S1) + 1 * (SES1H5N4F1S2 + SES1H5N5F1S2) + 0.333 * SES1H5N6F1S1 + 0.667 * (SES1H5N6F1S2 + SES1H5N6F2S2)
SES_tri_antennary	1 * (SES1H5N6F1 + SES1H5N6F2 + SES1H5N6F1S1 + SES1H5N6F1S2 + SES1H5N6F2S2)
SES_sialylation_per_galactose	SES_sialylation / SES_galactosylation * 100
LSL_bisection	1 * (LSL1H3N5 + LSL1H4N5 + LSL1H4N5S1 + LSL1H5N5S1)
LSL_galactosylation	0.5 * (LSL1H4N5 + LSL1H4N5S1) + 1 * (LSL1H5N4S1 + LSL1H5N4S2 + LSL1H5N5S1)
LSL_sialylation	0.5 * (LSL1H4N5S1 + LSL1H5N4S1 + LSL1H5N5S1) + 1 * LSL1H5N4S2
LSL_sialylation_per_galactose	LSL_sialylation / LSL_galactosylation * 100
TPL_fucosylation	1 * (TPL1H4N5F1S1 + TPL1H5N4F1S1 + TPL1H5N4F1S2 + TPL1H5N5F1S1 + TPL1H5N5F1S2 + TPL1H6N5F1S1)
TPL_bisection	1 * (TPL1H4N5F1S1 + TPL1H5N5S1 + TPL1H5N5F1S1 + TPL1H5N5F1S2)
TPL_galactosylation	0.5 * TPL1H4N5F1S1 + 1 * (TPL1H5N4F1S1 + TPL1H5N4F1S2 + TPL1H5N5S1 + TPL1H5N5F1S1 + TPL1H5N5F1S2 + TPL1H6N5F1S1)
TPL_sialylation	0.5 * (TPL1H4N5F1S1 + TPL1H5N4F1S1 + TPL1H5N5S1 + TPL1H5N5F1S1) + 1 * (TPL1H5N4F1S2 + TPL1H5N5F1S2) + 0.333 * TPL1H6N5F1S1
TPL_sialylation_per_galactose	TPL_sialylation / TPL_galactosylation * 100
HYT_sialic_acids	(2 * (HYT1H2N3S2 + HYT1H2N4S2 + HYT1H3N3S2 + HYT1H3N4S2 + HYT1H3N5S2 + HYT1H3N6S2 + HYT1H4N4S2 + HYT1H4N5S2 + HYT1H4N6S2 + HYT1H5N5S2 + HYT1H5N6S2) + 1 * (HYT1H2N5S1 + HYT1H3N3S1 + HYT1H3N4S1 + HYT1H3N5S1 + HYT1H3N6S1 + HYT1H4N4S1 + HYT1H4N5S1 + HYT1H5N5S1) + 3 * (HYT1H3N3S3 + HYT1H3N4S3 +

	HYT1H3N5S3 + HYT1H3N6S3 + HYT1H4N4S3 + HYT1H4N5S3 + HYT1H4N6S3 + HYT1H5N5S3 + HYT1H5N6S3) + 4 * (HYT1H3N3S4 + HYT1H3N4S4 + HYT1H4N4S4 + HYT1H4N5S4 + HYT1H5N5S4 + HYT1H3N5S4) + 5 * HYT1H4N4S5) / 100
HYT_galactoses	(2 * (HYT1H2N3S2 + HYT1H2N4S2 + HYT1H2N5S1) + 3 * (HYT1H3N3S1 + HYT1H3N3S2 + HYT1H3N3S3 + HYT1H3N3S4 + HYT1H3N4 + HYT1H3N4S1 + HYT1H3N4S2 + HYT1H3N4S3 + HYT1H3N4S4 + HYT1H3N5 + HYT1H3N5S1 + HYT1H3N5S2 + HYT1H3N5S3 + HYT1H3N6S1 + HYT1H3N6S2 + HYT1H3N6S3 + HYT1H3N5S4) + 4 * (HYT1H4N4 + HYT1H4N4S1 + HYT1H4N4S2 + HYT1H4N4S3 + HYT1H4N4S4 + HYT1H4N4S5 + HYT1H4N5 + HYT1H4N5S1 + HYT1H4N5S2 + HYT1H4N5S3 + HYT1H4N5S4 + HYT1H4N6S2 + HYT1H4N6S3 + HYT1H4N6) + 5 * (HYT1H5N5 + HYT1H5N5S1 + HYT1H5N5S2 + HYT1H5N5S3 + HYT1H5N5S4 + HYT1H5N6S3 + HYT1H5N6S2)) / 100
HYT_galnacs	(3 * (HYT1H2N3S2 + HYT1H3N3S1 + HYT1H3N3S2 + HYT1H3N3S3 + HYT1H3N3S4) + 4 * (HYT1H2N4S2 + HYT1H3N4 + HYT1H3N4S1 + HYT1H3N4S2 + HYT1H3N4S3 + HYT1H3N4S4 + HYT1H4N4 + HYT1H4N4S1 + HYT1H4N4S2 + HYT1H4N4S3 + HYT1H4N4S4 + HYT1H4N4S5) + 5 * (HYT1H2N5S1 + HYT1H3N5 + HYT1H3N5S1 + HYT1H3N5S2 + HYT1H3N5S3 + HYT1H4N5 + HYT1H4N5S1 + HYT1H4N5S2 + HYT1H4N5S3 + HYT1H4N5S4 + HYT1H5N5 + HYT1H5N5S1 + HYT1H5N5S2 + HYT1H5N5S3 + HYT1H5N5S4 + HYT1H3N5S4) + 6 * (HYT1H3N6S1 + HYT1H3N6S2 + HYT1H3N6S3 + HYT1H4N6S2 + HYT1H4N6S3 + HYT1H5N6S3 + HYT1H4N6 + HYT1H5N6S2)) / 100
HYT_Tn_antigens	(1 * (HYT1H2N3S2 + HYT1H3N4 + HYT1H3N4S1 + HYT1H3N4S2 + HYT1H3N4S3 + HYT1H3N4S4 + HYT1H4N5 + HYT1H4N5S1 + HYT1H4N5S2 + HYT1H4N5S3 + HYT1H4N5S4 + HYT1H5N6S3 + HYT1H5N6S2) + 2 * (HYT1H2N4S2 + HYT1H3N5 + HYT1H3N5S1 + HYT1H3N5S2 + HYT1H3N5S3 + HYT1H4N6S2 + HYT1H4N6S3 + HYT1H4N6 + HYT1H3N5S4) + 3 * (HYT1H2N5S1 + HYT1H3N6S1 + HYT1H3N6S2 + HYT1H3N6S3)) / 100

HYT_T_antigens	(1 * (HYT1H2N5S1 + HYT1H3N3S2 + HYT1H3N4S2 + HYT1H3N5S2 + HYT1H3N6S2 + HYT1H4N4S3 + HYT1H4N5S3 + HYT1H4N6S3 + HYT1H5N5S4) + 2 * (HYT1H3N3S1 + HYT1H3N4S1 + HYT1H3N5S1 + HYT1H3N6S1 + HYT1H4N4S2 + HYT1H4N5S2 + HYT1H4N6S2 + HYT1H5N5S3 + HYT1H5N6S3) + 3 * (HYT1H3N4 + HYT1H3N5 + HYT1H4N4S1 + HYT1H4N5S1 + HYT1H5N5S2 + HYT1H5N6S2) + 4 * (HYT1H4N4 + HYT1H4N5 + HYT1H5N5S1 + HYT1H4N6) + 5 * HYT1H5N5) / 100
HYT_sT_antigens	(2 * (HYT1H2N3S2 + HYT1H2N4S2 + HYT1H3N3S2 + HYT1H3N4S2 + HYT1H3N5S2 + HYT1H3N6S2 + HYT1H4N4S2 + HYT1H4N5S2 + HYT1H4N6S2 + HYT1H5N5S2 + HYT1H5N6S2) + 1 * (HYT1H2N5S1 + HYT1H3N3S1 + HYT1H3N4S1 + HYT1H3N5S1 + HYT1H3N6S1 + HYT1H4N4S1 + HYT1H4N5S1 + HYT1H5N5S1) + 3 * (HYT1H3N3S3 + HYT1H3N3S4 + HYT1H3N4S3 + HYT1H3N4S4 + HYT1H3N5S3 + HYT1H3N6S3 + HYT1H4N4S3 + HYT1H4N5S3 + HYT1H4N6S3 + HYT1H5N5S3 + HYT1H5N6S3 + HYT1H3N5S4) + 4 * (HYT1H4N4S4 + HYT1H4N4S5 + HYT1H4N5S4 + HYT1H5N5S4)) / 100
HYT_disialylated_O_antigens	1 * (HYT1H3N3S4 + HYT1H3N4S4 + HYT1H4N4S5 + HYT1H3N5S4)
HYT_sialic_acids_per_galactose	HYT_sialic_acids / HYT_galactoses
HYT_galactoses_per_galnac	HYT_galactoses / HYT_galnacs
ENI_fucosylation	(1 * (ENI1H5N4F1S1 + ENI1H5N4F1S2)) / (ENI1H5N4F1S1 + ENI1H5N4F1S2 + ENI1H5N4S1 + ENI1H5N4S2 + ENI1H5N5S1) * 100
ENI_sialylation	(0.5 * (ENI1H5N4F1S1 + ENI1H5N4S1 + ENI1H5N5S1) + 1 * (ENI1H5N4F1S2 + ENI1H5N4S2)) / (ENI1H5N4F1S1 + ENI1H5N4F1S2 + ENI1H5N4S1 + ENI1H5N4S2 + ENI1H5N5S1) * 100
ENI_hybrid	1 * (ENI1H5N3F1S1 + ENI1H5N3S1)
LAGC_fucosylation	1 * (LAGC1H5N4F1S1 + LAGC1H5N4F1S2 + LAGC1H5N5F1S1 + LAGC1H5N5F1S2 + LAGC1H6N5F1S1 + LAGC1H6N5F1S2 + LAGC1H6N5F1S3 + LAGC1H6N6F1S2 + LAGC1H5N4F1S2Su1 + LAGC1H6N5F1S1Su1)

LAGC_bisection	1 * (LAGC1H5N5F1S1 + LAGC1H5N5F1S2 + LAGC1H5N6S2 + LAGC1H6N6F1S2)
LAGC_galactosylation	1 * (LAGC1H5N4F1S1 + LAGC1H5N4F1S2 + LAGC1H5N5F1S1 + LAGC1H5N5F1S2 + LAGC1H6N5F1S1 + LAGC1H6N5F1S2 + LAGC1H6N5F1S3 + LAGC1H6N6F1S2 + LAGC1H5N4F1S2Su1 + LAGC1H6N5F1S1Su1) + 0.667 * LAGC1H5N6S2
LAGC_sialylation	0.5 * (LAGC1H5N4F1S1 + LAGC1H5N5F1S1) + 1 * (LAGC1H5N4F1S2 + LAGC1H5N5F1S2 + LAGC1H6N5F1S3 + LAGC1H5N4F1S2Su1) + 0.333 * (LAGC1H6N5F1S1 + LAGC1H6N5F1S1Su1) + 0.667 * (LAGC1H6N5F1S2 + LAGC1H5N6S2 + LAGC1H6N6F1S2)
LAGC_tri_antennary	1 * (LAGC1H6N5F1S1 + LAGC1H6N5F1S2 + LAGC1H6N5F1S3 + LAGC1H5N6S2 + LAGC1H6N6F1S2 + LAGC1H6N5F1S1Su1)
LAGY_fucosylation	(1 * (LAGY1H4N5F1 + LAGY1H5N4F1S1 + LAGY1H5N4F1S2 + LAGY1H5N5F1 + LAGY1H5N5F1S1 + LAGY1H5N5F1S2 + LAGY1H6N5F1S1 + LAGY1H6N5F1S2 + LAGY1H5N4F1S2Su1 + LAGY1H5N5F1S2Su1 + LAGY1H6N5F1S2Su1 + LAGY1H6N6F1S2Su1)) / (LAGY1H4N5F1 + LAGY1H5N4S1 + LAGY1H5N4S2 + LAGY1H5N4F1S1 + LAGY1H5N4F1S2 + LAGY1H5N5F1 + LAGY1H5N5F1S1 + LAGY1H5N5F1S2 + LAGY1H6N5F1S1 + LAGY1H6N5F1S2 + LAGY1H5N4F1S2Su1 + LAGY1H5N5F1S2Su1 + LAGY1H6N5F1S2Su1 + LAGY1H6N6F1S2Su1) * 100
LAGY_bisection	(1 * (LAGY1H4N5F1 + LAGY1H5N5F1 + LAGY1H5N5F1S1 + LAGY1H5N5F1S2 + LAGY1H5N5F1S2Su1 + LAGY1H6N6F1S2Su1)) / (LAGY1H4N5F1 + LAGY1H5N4S1 + LAGY1H5N4S2 + LAGY1H5N4F1S1 + LAGY1H5N4F1S2 + LAGY1H5N5F1 + LAGY1H5N5F1S1 + LAGY1H5N5F1S2 + LAGY1H6N5F1S1 + LAGY1H6N5F1S2 + LAGY1H5N4F1S2Su1 + LAGY1H5N5F1S2Su1 + LAGY1H6N5F1S2Su1 + LAGY1H6N6F1S2Su1) * 100
LAGY_galactosylation	(0.5 * LAGY1H4N5F1 + 1 * (LAGY1H5N4S1 + LAGY1H5N4S2 + LAGY1H5N4F1S1 + LAGY1H5N4F1S2 + LAGY1H5N5F1 + LAGY1H5N5F1S1 + LAGY1H5N5F1S2 + LAGY1H6N5F1S1 + LAGY1H6N5F1S2 + LAGY1H5N4F1S2Su1 + LAGY1H5N5F1S2Su1 + LAGY1H6N5F1S2Su1 + LAGY1H6N6F1S2Su1)) / (LAGY1H4N5F1 + LAGY1H5N4S1 +

	LAGY1H5N4S2 + LAGY1H5N4F1S1 + LAGY1H5N4F1S2 + LAGY1H5N5F1 + LAGY1H5N5F1S1 + LAGY1H5N5F1S2 + LAGY1H6N5F1S1 + LAGY1H6N5F1S2 + LAGY1H5N4F1S2Su1 + LAGY1H5N5F1S2Su1 + LAGY1H6N5F1S2Su1 + LAGY1H6N6F1S2Su1) * 100
LAGY_sialylation	(0.5 * (LAGY1H5N4S1 + LAGY1H5N4F1S1 + LAGY1H5N5F1S1) + 1 * (LAGY1H5N4S2 + LAGY1H5N4F1S2 + LAGY1H5N5F1S2 + LAGY1H5N4F1S2Su1 + LAGY1H5N5F1S2Su1) + 0.333 * LAGY1H6N5F1S1 + 0.667 * (LAGY1H6N5F1S2 + LAGY1H6N5F1S2Su1 + LAGY1H6N6F1S2Su1)) / (LAGY1H4N5F1 + LAGY1H5N4S1 + LAGY1H5N4S2 + LAGY1H5N4F1S1 + LAGY1H5N4F1S2 + LAGY1H5N5F1 + LAGY1H5N5F1S1 + LAGY1H5N5F1S2 + LAGY1H6N5F1S1 + LAGY1H6N5F1S2 + LAGY1H5N4F1S2Su1 + LAGY1H5N5F1S2Su1 + LAGY1H6N5F1S2Su1 + LAGY1H6N6F1S2Su1) * 100
LAGY_tri_antennary	(1 * (LAGY1H6N5F1S1 + LAGY1H6N5F1S2 + LAGY1H6N5F1S2Su1 + LAGY1H6N6F1S2Su1)) / (LAGY1H4N5F1 + LAGY1H5N4S1 + LAGY1H5N4S2 + LAGY1H5N4F1S1 + LAGY1H5N4F1S2 + LAGY1H5N5F1 + LAGY1H5N5F1S1 + LAGY1H5N5F1S2 + LAGY1H6N5F1S1 + LAGY1H6N5F1S2 + LAGY1H5N4F1S2Su1 + LAGY1H5N5F1S2Su1 + LAGY1H6N5F1S2Su1 + LAGY1H6N6F1S2Su1) * 100
LAGC_sialylation_per_galactose	LAGC_sialylation / LAGC_galactosylation * 100
LAGY_sialylation_per_galactose	LAGY_sialylation / LAGY_galactosylation * 100

Table S2. Summary of coexisting placenta-related pregnancy complications.

Complication	N patients
Preeclampsia alone	2
Preeclampsia with preterm birth and SGA	3
Preeclampsia with preterm birth without SGA	2
Pregnancy-induced hypertension alone	12
Pregnancy-induced hypertension with preterm birth without SGA	1
Pregnancy-induced hypertension without preterm birth with SGA	2
Preterm birth alone	12
Preterm birth with SGA	6
SGA alone	14

Supplemental Figures

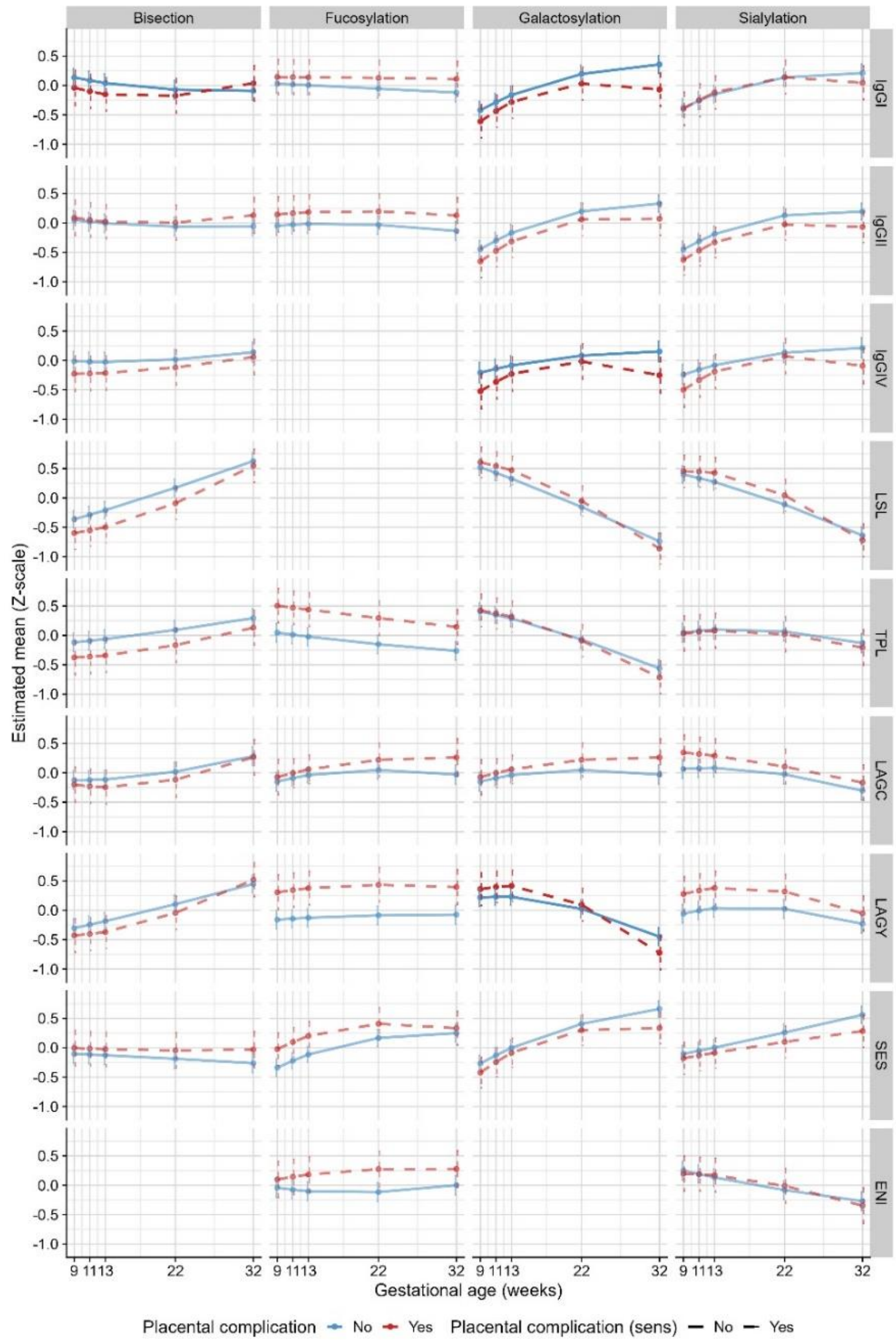

Figure S1: Trajectories of maternal IgG and IgA glycosylation traits across gestation in a sensitivity analysis, in which spontaneous preterm birth without other placenta-related complications was reclassified as not a placenta-related complication. *Lines represent estimated marginal means (Z-standardized values) for pregnancies with placenta-related complications (red, dashed) and without complications (blue, solid), derived from linear mixed-effects models. Each column facet represents a glycosylation trait (fucosylation, bisection, galactosylation, or sialylation), and each row facet represents an individual glycosylation site. Error bars indicate 95% confidence intervals of the estimated marginal means. More intense line colors denote traits with a statistically significant interaction term (placenta complication × gestational age) in the mixed model, while lighter colors indicate non-significant interactions.*

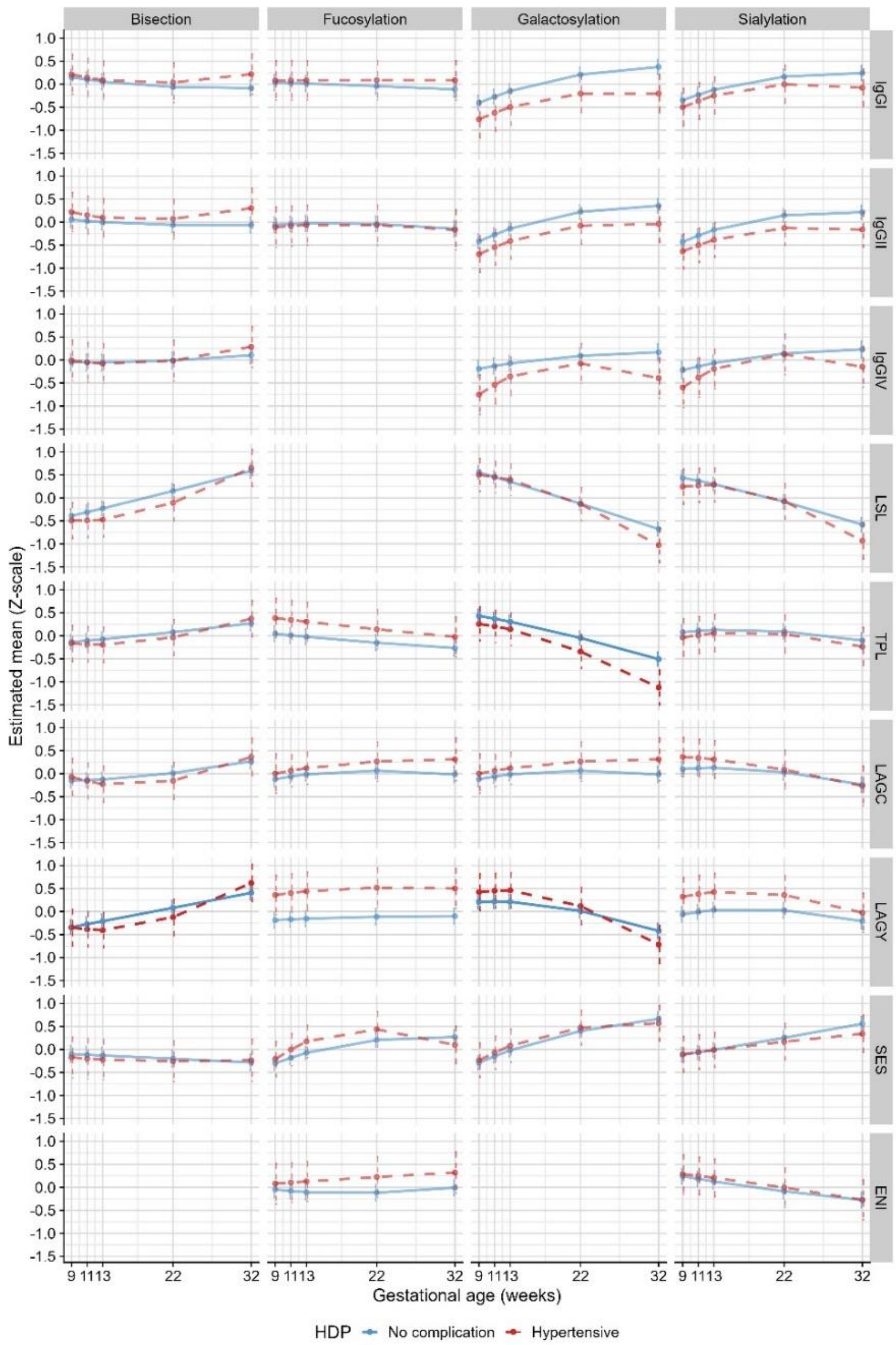

Figure S2: Trajectories of maternal IgG and IgA glycosylation traits across gestation in a sensitivity analysis, comparing hypertensive disorders of pregnancy with uncomplicated pregnancies. *Lines represent estimated marginal means (Z-standardized values) for pregnancies with pre-eclampsia or gestational hypertension (red, dashed) and without any placenta-related complication (blue, solid), derived from linear mixed-effects models. Each column facet represents a glycosylation trait (fucosylation, bisection, galactosylation, or sialylation), and each row facet represents an individual glycosylation site. Error bars indicate 95% confidence intervals of the estimated marginal means. More intense line colors denote traits with a statistically significant interaction term (placenta complication × gestational age) in the mixed model, while lighter colors indicate non-significant interactions.*

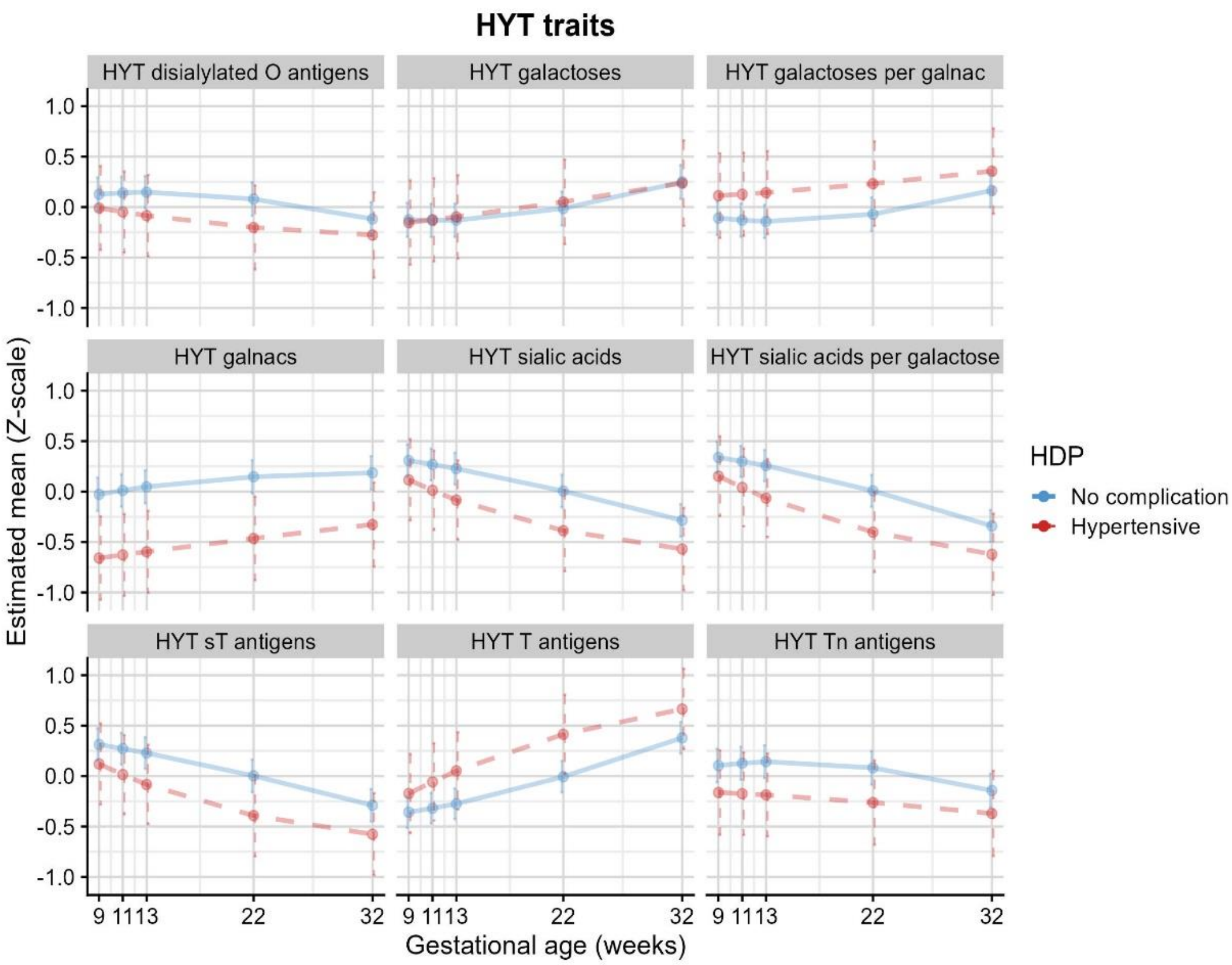

Figure S3: Estimated trajectories of IgA1 O-glycosylation (HYT region) across gestation in pregnancies with hypertensive disorders, compared to those without placenta-related complications. *Lines represent estimated marginal means (Z-standardized values) for women with hypertensive disorders (red, dashed) and uncomplicated pregnancies (blue, solid), derived from linear mixed-effects models. Error bars denote 95% confidence intervals of the estimated marginal means.*
